# Differential distribution of plasma apoA-I and apoB levels and clinical significance of apoB/apoA-I ratio in ischemic stroke subtypes

**DOI:** 10.3389/fneur.2024.1398830

**Published:** 2024-06-24

**Authors:** Nguyen Van Tuyen, Nguyen Hoang Ngoc, Phan Quoc Hoan, Nguyen Thi Yen, Nghiem Xuan Hoan, Nguyen Cam Thach

**Affiliations:** ^1^Department of Stroke, Institute of Neurology, 108 Institute of Clinical Medical and Pharmaceutical Sciences, Hanoi, Vietnam; ^2^VNU University of Medicine and Pharmacy, Hanoi, Vietnam; ^3^Department of Molecular Biology, 108 Institute of Clinical Medical and Pharmaceutical Sciences, Hanoi, Vietnam; ^4^Department of Biochemistry, 108 Institute of Clinical Medical and Pharmaceutical Sciences, Hanoi, Vietnam; ^5^Vietnamese-German Center for Medical Research (VG-CARE), Hanoi, Vietnam

**Keywords:** apoB, apoA-I, ischemic stroke, intracranial atherosclerosis, extracranial atherosclerosis, small artery occlusion, apoB/apoA-I ratio

## Abstract

**Background and purpose:**

Ischemic stroke (IS) is classified into clinical subtypes and likely influenced by various lipid components. Nevertheless, the roles of apolipoprotein A-I (apoA-I), apolipoprotein B (apoB), and apoB/apoA-I ratio in different IS subtypes remain underexplored. This study aimed to investigate the differential distribution of plasma apoA-I and apoB levels among IS subtypes and to evaluate the predictive value of the apoB/apoA-I ratio in assessing IS subtypes and disease severity.

**Methods:**

In this study, 406 IS patients were categorized into three IS-subtypes based on clinical manifestations and imaging assessment, including intracranial atherosclerosis-related IS patients (ICAS, *n* = 193), extracranial atherosclerosis-related IS patients (ECAS, *n* = 111), and small artery occlusion-related IS patients (SAO, *n* = 102). Plasma apoA-I and apoB levels were measured upon hospital admission. Random forest (RF) models were performed to assess predictive values of these apolipoproteins apoB, apoA-I and their ratio in assessing IS subtype stratification and disease severity.

**Results:**

Serum apoA-I levels were significantly lower in ICAS compared to ECAS and SAO patients (*p* < 0.0001), while apoB levels were higher in ICAS patients (*p* < 0.0001). The apoB/apoA-I ratio was significantly higher in ICAS compared to ECAS and SAO patients (*p* < 0.0001). Correlation analyses found a significant correlation between the apoB/apoA-I ratio and conventional lipid components. Additionally, RF models and plots of variable importance and distribution of minimal depth revealed that the apoB/apoA-I ratio played the most influential predictor in predicting IS subtypes and stenosis severity.

**Conclusion:**

Our study shows the differential distribution of apoA-I and apoB IS subtypes and reveals the significance of the apoB/apoA-I ratio in assessing IS subtypes and arterial stenosis severity. Further studies are warranted to validate these findings and enhance their clinical applicability.

## Introduction

Globally, stroke stands as the second leading cause of mortality globally, contributing to 11.6% of all deaths (1). In which, ischemic stroke (IS) prevails as the most prevalent, constituting 62.4% of all stroke incidents worldwide in 2019 (1, 2). Despite significant advances in treatment and therapy for IS, mortality rates remain high. The critical factor is the time elapsed between symptom onset and seeking medical attention. This underscores the importance of swift action in stroke management. Among survivors, the recurrence rate of stroke patients after the acute event is very high, with approximately 15–30% at risk within the first 2 years, 25% within 5 years, and doubling after 10 years (3). Importantly, IS can inflict profound neurological impairment and persistent disability in these survival individuals, thereby imposing significant health and economic burdens on societies.

Ischemic stroke is a vascular disorder of the brain’s circulatory system, with multiple causes leading to this condition, such as arterial atherosclerosis, cardiac emboli or blood clots originating from the heart, and coagulation disorders. Among which arterial atherosclerosis is a known strong risk factor for IS (4). Arterial atherosclerosis can occur both intracranially and extracranially, with intracranial atherosclerosis (ICAS) is more prevalent in Asian patients and is associated with a high risk of recurrence, whereas extracranial atherosclerosis (ECAS) is more common in individuals from western countries (4, 5). Meanwhile, a distinct IS subtype, known as small-artery occlusion (SAO), resulting in small (<15 mm in axial diameter) subcortical infarcts, is well categorized by the Trial of ORG10172 in Acute Stroke Treatment (TOAST classification) (6). This subtype is prevalent in developing nations, particularly accounting for 27.3% of IS cases in China (7, 8).

Atherosclerosis is a chronic lipid-driven and maladaptive inflammatory disease of arterial intima. Several factors were considered to contribute to pathogenesis of artery atherosclerosis formation. It is characterized by the dysfunction of lipid homeostasis and signaling pathways that control the inflammation. High-risk factors, such as hypertension, diabetes, and smoking, cause vascular endothelial dysfunction and increased permeability. This leads to accumulation of cholesterol-containing low-density lipoproteins (LDL) in the intima, which initiates a complex series of inflammatory and biochemical reactions involving accumulation of extracellular matrix, activation of the endothelium, infiltration of monocytes and T cells, intimal thickening, fibrous cap formation, and angiogenesis (9–11).

In clinical practice, one of the important measures in the prevention and treatment of arterial atherosclerotic disease is the control of lipoproteins (cholesterol, HDL, LDL). These are commonly measured parameters that help clinicians assess metabolic disorders and identify risk factors for arterial atherosclerosis (12–14). Nonetheless, apolipoproteins, in particular, apolipoprotein B (apoB) may be more useful clinically than LDL cholesterol because it captures greater information about atherogenic particles and is not influenced by heterogeneity of particle cholesterol content (15). Apolipoproteins are integral components of lipoproteins and play essential roles in lipid metabolism and transport. Unlike lipoproteins, apolipoproteins are directly involved in the pathophysiology of atherosclerosis. ApoB is present on atherogenic lipoproteins (such as LDL and VLDL) and is directly involved in their interaction with arterial endothelial cells, promoting the formation of atherosclerotic plaques. Additionally, apolipoprotein A-I (apoA-I), the major protein component of HDL particles, is associated with reverse cholesterol transport and has protective effects against atherosclerosis. In recent years, several investigations have shed light on the significance of apoA-I, apoB, and the apoB/apoA-I ratio in predicting and assessing susceptibility to cardiovascular diseases (16–20). These metrics serve as indicators of the balance between atherogenic and anti-atherogenic lipoproteins, offering insights into the status of arterial atherosclerotic disease.

While apoA-I and apoB hold promise as biomarkers, potentially traditional lipoproteins in terms of enhanced accuracy and practicality in predicting arterial atherosclerosis in cardiovascular diseases. However, to date, research on apolipoproteins and their application in monitoring, prognostication, and preventive treatment of IS has garnered scant attention. To the best of our knowledge, only a few studies have investigated apolipoproteins in IS patients (21–23). Therefore, exploring the potential roles of apoA-I and apoB in IS patients holds significant value, particularly in the Vietnamese population where IS represents a substantial medical concern. In addition to these reasons, we conducted this study aimed to investigate the differential distribution of plasma apoA-I and apoB levels among IS subtypes and to evaluate the predictive value of the apoB/apoA-I ratio in assessing IS subtypes and disease severity.

## Subjects and methods

### Study subjects

Between March 2019 and September 2020, patients presenting with acute cerebral ischemia were referred to the Stroke Centre of the 108 Military Central Hospital for examination and clinical management. Acute ischemic stroke diagnosis relies on clinical manifestations and imaging findings from magnetic resonance angiography (MRI) or computed tomography (CT) scans of the brain. Meanwhile, the diagnosis of narrowed or blocked blood vessels relies on magnetic resonance angiography (MRA) or digital subtraction angiography (DSA). During study period, we enrolled 406 patients with acute IS who met the following criteria: aged ≥18 years old and presenting with acute IS symptoms within 2 days of onset. Patients were excluded if they exhibited the following characteristics: (1) IS associated with valvular heart disease, cardiac arrhythmias, or complete atrioventricular block as detected on electrocardiography; (2) IS patients with implanted pacemakers; (3) IS resulting from conditions associated with increased blood clotting, such as sickle cell anemia or systemic lupus erythematosus; (4) patients with comorbidities including heart failure, renal failure, liver cirrhosis, or thyroid cancer; (5) patients taking medications known to affect laboratory test results lipid profiling, such as carbamazepine, estrogen, lovastatin, simvastatin, phenobarbital, or pravastatin. In addition, we had 14 IS patients with stenosis in both intracranial and extracranial large arteries. Due to the small number of patients compared to the other groups, we have excluded these cases from the analyses.

### Data collection and patient classification

Upon admission, comprehensive baseline data encompassing age, gender, medical history, and physical examination findings were collected. Each patient underwent thorough clinical assessment, neurological examination, pertinent laboratory investigations, cardiac assessment, and evaluation of imaging methodologies. Lipid profiling was conducted in accordance with established guidelines, with criteria including low levels of HDL-C (<1.03 mmol/L in men, 1.30 mmol/L in women), elevated total cholesterol (≥5.2 mmol/L), increased triglyceride levels (TG ≥ 1.7 mmol/L), and nonoptimal LDL-C values (≥2.6 mmol/L) (24).

The enrolled patients were stratified into two distinct cohorts using the TOAST classification (6, 25). In brief, group A comprised patients diagnosed with IS resulting from large artery atherosclerosis. Imaging assessments revealed cortical or subcortical infarcts, or hemispheric infarcts exceeding 1.5 cm in diameter, accompanied by evidence of greater than 50% stenosis in the affected arteries. This group was further divided into two subgroups: the first subgroup consisted of patients with intracranial atherosclerosis (ICAS, *n* = 193), while the second subgroup consisted of patients with extracranial atherosclerosis (ECAS, *n* = 111). Group B consisted of IS patients attributed to small artery occlusion (SAO, *n* = 102).

The degree of stenosis was assessed using the methodology outlined in the study conducted by Samuels OB et al. (26). It was calculated using the formula: degree of stenosis (%) = (1 – diameter at the narrowest point of the narrow segment/diameter of the proximal normal vessel) × 100%. Patients in this study were categorized into three groups based on the degree of stenosis: mild stenosis group (stenosis ≤49%), moderate–severe stenosis group (stenosis between 60 and 90%), and an extreme stenosis group (stenosis >90% or occlusion).

### Blood sampling and laboratory analyses

Blood samples from each patient were collected at hospital admission. The samples were centrifuged according to the standard protocol to separate plasma from blood cells. Subsequently, conventional lipid profile, including triglycerides, total cholesterol, HDL, and LDL levels, was routinely measured on the AU5800 Beckman Coulter Analyzers (Beckman Coulter), employing a quantitative photometric method based on the principles of light absorbance measurement by chemical substances in solution.

Following the routine measurement of conventional lipids, apoA-I and apoB were quantified based on the immunoturbidimetric method. This process was carried out on the AU5800 analyzer according to the manufacturer’s instructions provided by Beckman Coulter. In this method, the sample or standard was mixed with a reagent containing anti-apoA-I or apoB antibodies. Upon binding with these antibodies, apoA-I or apoB in the sample formed a precipitated complex. The turbidity of this complex was then measured at a wavelength of 340 nm. The level of turbidity observed was directly proportional to the concentration of apoA-I or apoB present in the clinical sample.

### Statistical analyses

Statistical analyses were conducted using R version 4.3.2.^1^ Chi-square, Kruskal-Wallis, and Mann–Whitney-Wilcoxon tests were employed to compare group differences in qualitative or quantitative variables as appropriate. Spearman’s rank correlation test was applied to evaluate correlations between apoA-I, apoB, and other laboratory parameters. Random forest models were utilized to assess the potential predictor, including apoA-I, apoB, apoB/apoA-I ratio, and other independent factors for IS subtypes and arterial stenosis severity. Receiver operating characteristic (ROC) curves generated from random forest (RF) models assessed the predictive value of variables of interest in discriminating clinical status by calculating the area under the ROC curve (AUC). We analyzed the variable importance and distribution of minimal depth through tree modeling to determine the most influential variable(s) in predicting the outcome. All statistical significance was defined as a two-sided *p*-value of <0.05.

## Results

### Baseline characteristics of participants

**Figure 1:**
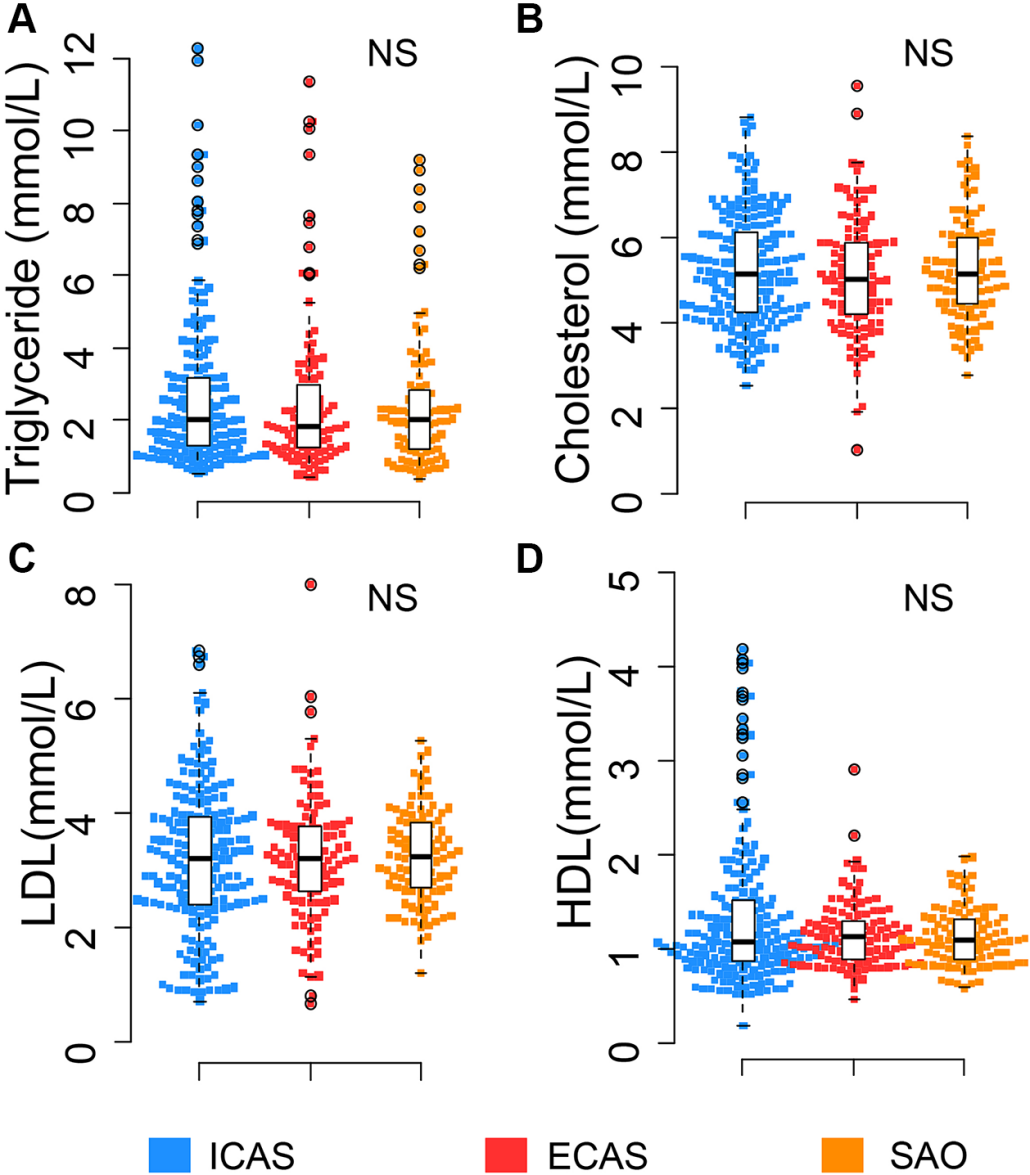
Distribution of routine lipid profile in IS patients: **(A)** Triglyceride; **(B)** Cholesterol; **(C)** LDL; **(D)** HDL. Box-plots illustrate median values with range (min-max) and outliers; NS, not significant; ICAS, intracranial atherosclerosis; ECAS, extracranial atherosclerosis; SAO, small artery occlusion; NICAS, Non-ICAS (ECAS+SAO). *p*-values were calculated by Kruskal – Wallis tests.

**Figure 2:**
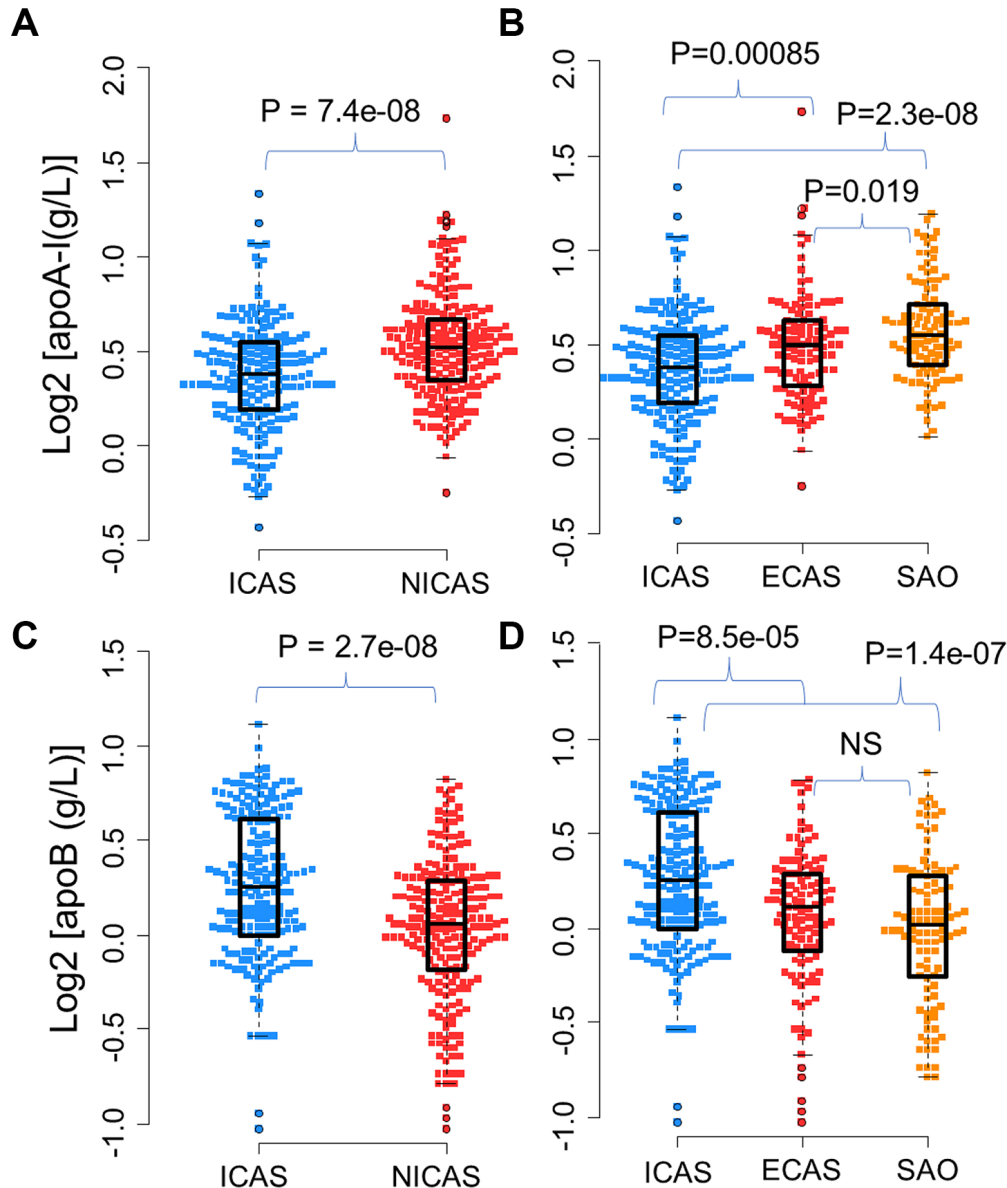
Distribution of apoA-I and apoB levels in IS patients: **(A)** Distribution of apoA-I in ICAS and NICAS; **(B)** Distribution of apoA-I in ICAS, ECAS and SAO; **(C)** Distribution of apoB in ICAS and NICAS; **(D)** Distribution of apoB in ICAS, ECAS and SAO. Box-plots illustrate median values with range (min-max) and outliers; NS, not significant; ICAS, intracranial atherosclerosis; ECAS, extracranial atherosclerosis; SAO, small artery occlusion; NICAS, Non-ICAS (ECAS+SAO). *p*-values were calculated by Wilcoxon tests.

**Figure 3:**
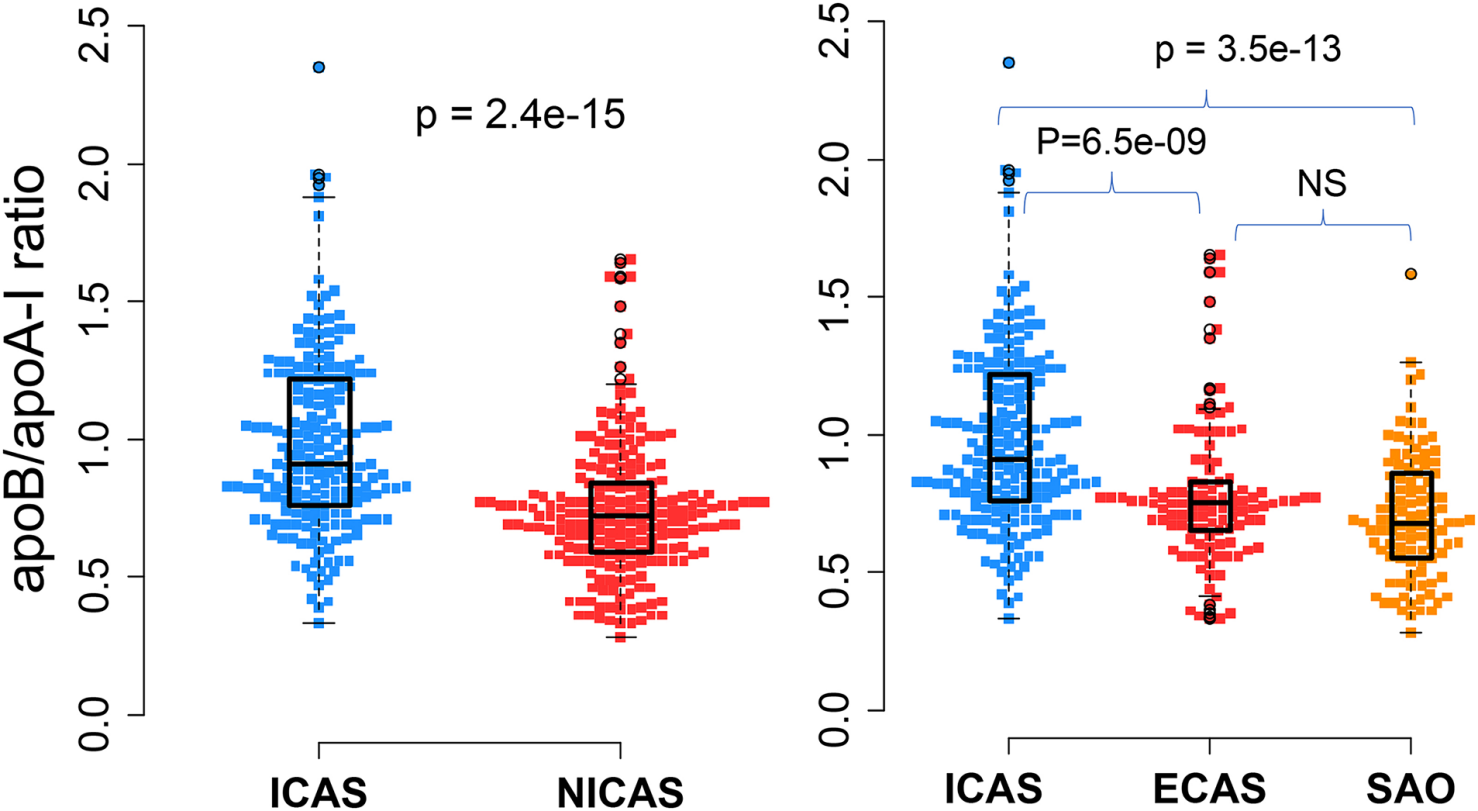
Association of apoB/apoA-I ratio with IS patients according to the TOAST classification system: Box-plots illustrate median values with range (min-max) and outliers; NS, not significant; ICAS, intracranial atherosclerosis; ECAS, extracranial atherosclerosis; SAO, small artery occlusion; NICAS, Non-ICAS (ECAS+SAO). *p*-values were calculated by Wilcoxon tests.

The baseline profile of 406 consecutive IS patients who met the inclusion criteria enrolled in this study were described in Table 1 and Figures 1–3. In this cohort, the median age at diagnosis in IS subgroups was 65–66 years [ICAS: 65 (18–90); ECAS: 66 (34–93); SAO: 65 (28–86); *p* > 0.05]. Most of the patients were male (>75% for each group). SAO patient group had a higher percentile (21.6%) of BMI ≥ 25 compared to IS patients with large artery atherosclerosis (ICAS: 8.8% and ECAS:7.2%). There was no significant difference in the distribution of comorbidities, including hypertension and diabetes mellitus, among the groups. There was no difference in the levels of blood lipid types such as total cholesterol, LDL, triglyceride, and HDL between the groups as seen in Table 1 and Figure 1.

**Table 1:** Subjects’ characteristics at baseline.

Clinical characteristics		ICAS (*n* = 193)		ECAS (*n* = 111)		SAO (*n* = 102)		*p*-value
Age (years)		65	18–90	66	34–93	65	28–86	0.56
Male/Female, *n* (%)		145/193	75%	90/111	81%	78/102	76%	0.48
BMI classification	<18.5	7	3.6%	2	1.8%	6	5.9%	0.002
	18.5–25	169	87.6%	101	91.0%	74	72.5%	
	>25	17	8.8%	8	7.2%	22	21.6%	
Hypertension *n* (%)	Yes	134	69.4%	74	66.7%	70	68.6%	0.88
	No	59	30.6%	37	33.3%	32	31.4%	
Diabetes *n* (%)	Yes	42	21.8%	27	24.3%	15	14.7%	0.19
	No	151	78.2%	84	75.7%	87	85.3%	
**Lipid profile**								
Cholesterol (μmol/L)		5.23 ± 1.31		5.08 ± 1.37		5.24 ± 1.20		0.65
LDL (μmol/L)		3.17 ± 1.09		3.2 ± 1.09		3.24 ± 0.8		0.81
Triglyceride (μmol/L)		2.7 ± 2.12		2.5 ± 2.11		2.44 ± 1.86		0.43
HDL (μmol/L)		1.32 ± 0.77		1.15 ± 0.36		1.13 ± 0.31		0.91
ApoA1 (g/L)		1.32 ± 0.27		1.43 ± 0.32		1.51 ± 0.28		4.37E-08
ApoB (g/L)		1.24 ± 0.32		1.07 ± 0.26		1.03 ± 0.27		7.28E-08
ApoB/A1 ratio		0.98 ± 0.33		0.78 ± 0.26		0.7 ± 0.23		4.52E-15

Values of lipid profile given are presented as mean ± SD; *p*-values were calculated by Chi-squared test and Kruskal-Wallis test where appropriate.

The distribution of apoA-I and apoB levels upon hospital admission among subgroups of IS patients was illustrated in Table 1 and Figure 2. Serum apoA-I levels were significantly lower in patients with ICAS compared to those with ECAS and SAO [mean ± SD: ICAS (1.32 ± 0.27 g/L); ECAS (1.43 ± 0.32); SAO (1.51 ± 0.28 g/L) (*p* < 0.0001), Figures 2A,B]. Conversely, serum apoB levels were higher in ICAS patients [(mean ± SD: 1.24 ± 0.32 g/L) followed by ECAS (mean ± SD: 1.07 ± 0.26 g/L) and SAO patients (mean ± SD: 1.03 ± 0.27 g/L) (*p* < 0.0001), Figures 2C,D]. These differences persisted with a consistent trend when comparing apoA-I and apoB levels between the ICAS group and the non-ICAS group (comprising ECAS and SAO) (*p* < 0.0001).

As presented in Table 1 and Figure 3, the apoB/apoA-I ratio exhibited a remarkably higher value in patients with intracranial arterial stenosis (ICAS) compared to those without intracranial arterial stenosis (NICAS) (*p* = 1.4e-15). Furthermore, upon comparison across subgroups, it was evident that ICAS patients had a significantly higher apoB/apoA-I ratio compared to patients with extracranial arterial stenosis (ECAS) and small artery occlusion (SAO) [mean ± SD: ICAS (0.98 ± 0.33); ECAS (0.78 ± 0.26); SAO (0.7 ± 0.23) (*p* < 0.0001)]. Conversely, no significant difference was observed between ECAS and SAO patients (*p* > 0.05).

### Correlation between apoA-I, apoB levels, and apoB/apoA-I ratio with routine lipids in IS patients

**Figure 4:**
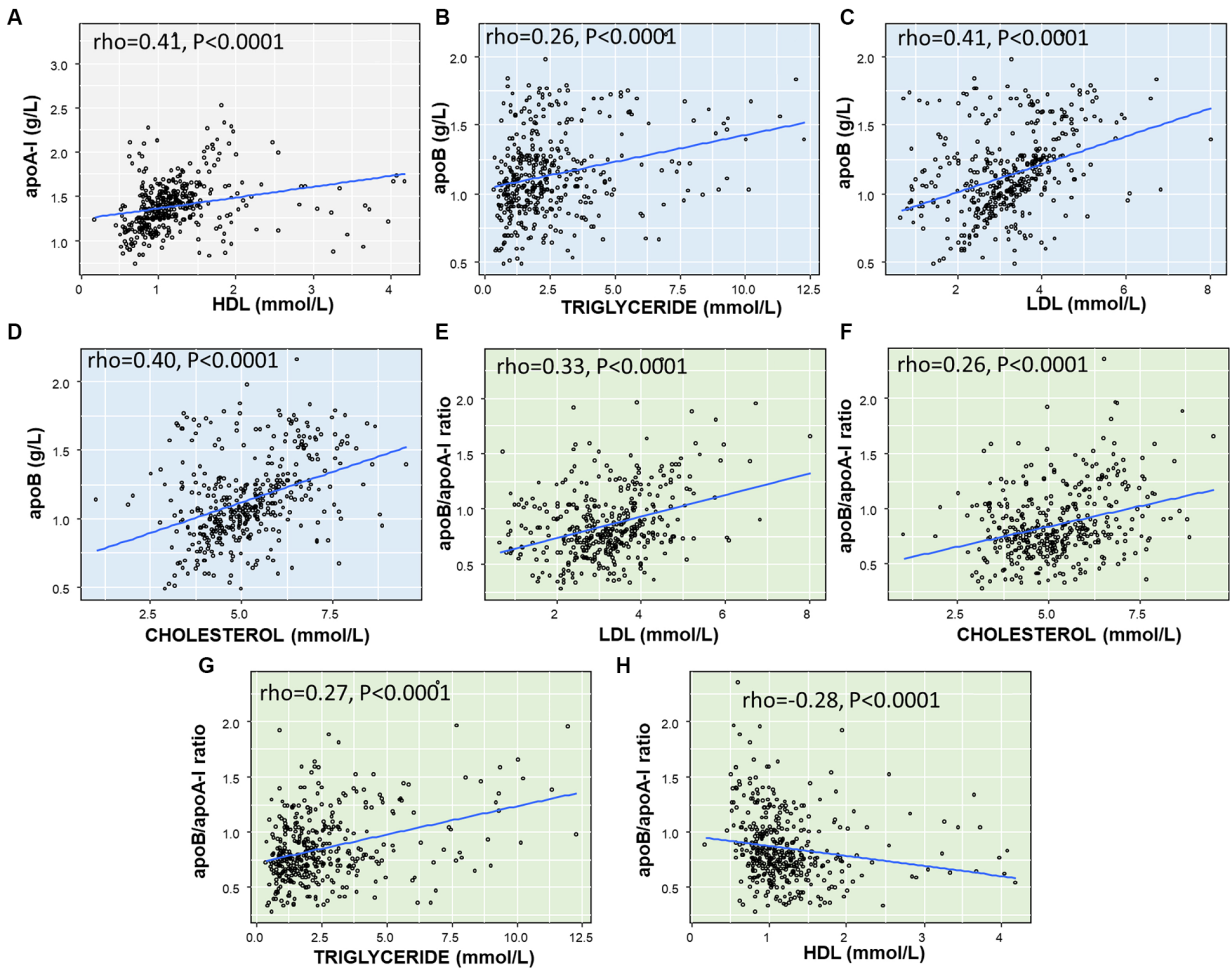
Correlation between apoA-I, apoB, apoB/apoA-I ratio with conventional lipid components in IS patients: **(A)** correlation between ApoA-I and HDL; **(B)** correlation between ApoB and Triglyceride; **(C)** correlation between ApoB and LDL; **(D)** Correlation between ApoB and Cholesterol; **(E)** Correlation between apoB/apoA-I ratio and LDL; **(F)** Correlation between apoB/apoA-I ratio and Cholesterol; **(G)** correlation between apoB/apoA-I ratio and Triglyceride; **(H)** correlation between apoB/apoA-I ratio and HDL. The correlation coefficient between two variables was calculated by using spearman’s rank correlation coefficient. Spearman’s rho (rho) and *p*-value are given.

Analysis of the correlation between apoA-I, apoB levels, and the apoB/apoA-I ratio with routine lipid profile indices, including total cholesterol, LDL, HDL, and triglycerides, we found that apoA-I was significantly correlated with HDL (rho = 0.41, *p* < 0.0001, Figure 4A), while there was either no correlation or weak correlation with the other lipid indices (data not presented in Figure 4). As for apoB, there was a correlation with triglyceride (rho = 0.26, *p* < 0.0001, Figure 4B), LDL (rho = 0.41, *p* < 0.0001, Figure 4C), total cholesterol (rho = 0.40, *p* < 0.0001, Figure 4D), and but no correlation with HDL (not presented). Regarding the apoB/apoA-I ratio, the analyses showed a positive correlation with LDL (rho = 0.33, *p* < 0.0001, Figure 4E), total cholesterol (rho = 0.26, *p* < 0.0001, Figure 4F), triglycerides (rho = 0.27, *p* < 0.0001, Figure 4G), and a negative correlation with HDL (rho = −0.28, *p* < 0.0001, Figure 4H).

In addition, we analyzed the apo-AI, apoB, and apoB/apoA-I ratio profiles in a subset of 25 cases with markedly high triglyceride levels (>6 mmol/L). The results showed that the majority of patients (22 out of 25, 88%) had low levels of apo-AI, defined as less than 1.6 g/L. (27) Moreover, elevated levels of apoB and an increased apoB/apoA-I ratio (normal range: apoB <1.3 g/L; apoB/apoA-I ratio < 0.77), were observed in 48% (12 out of 25) and 68% (17 out of 25) of the patients, respectively.

**Figure 5:**
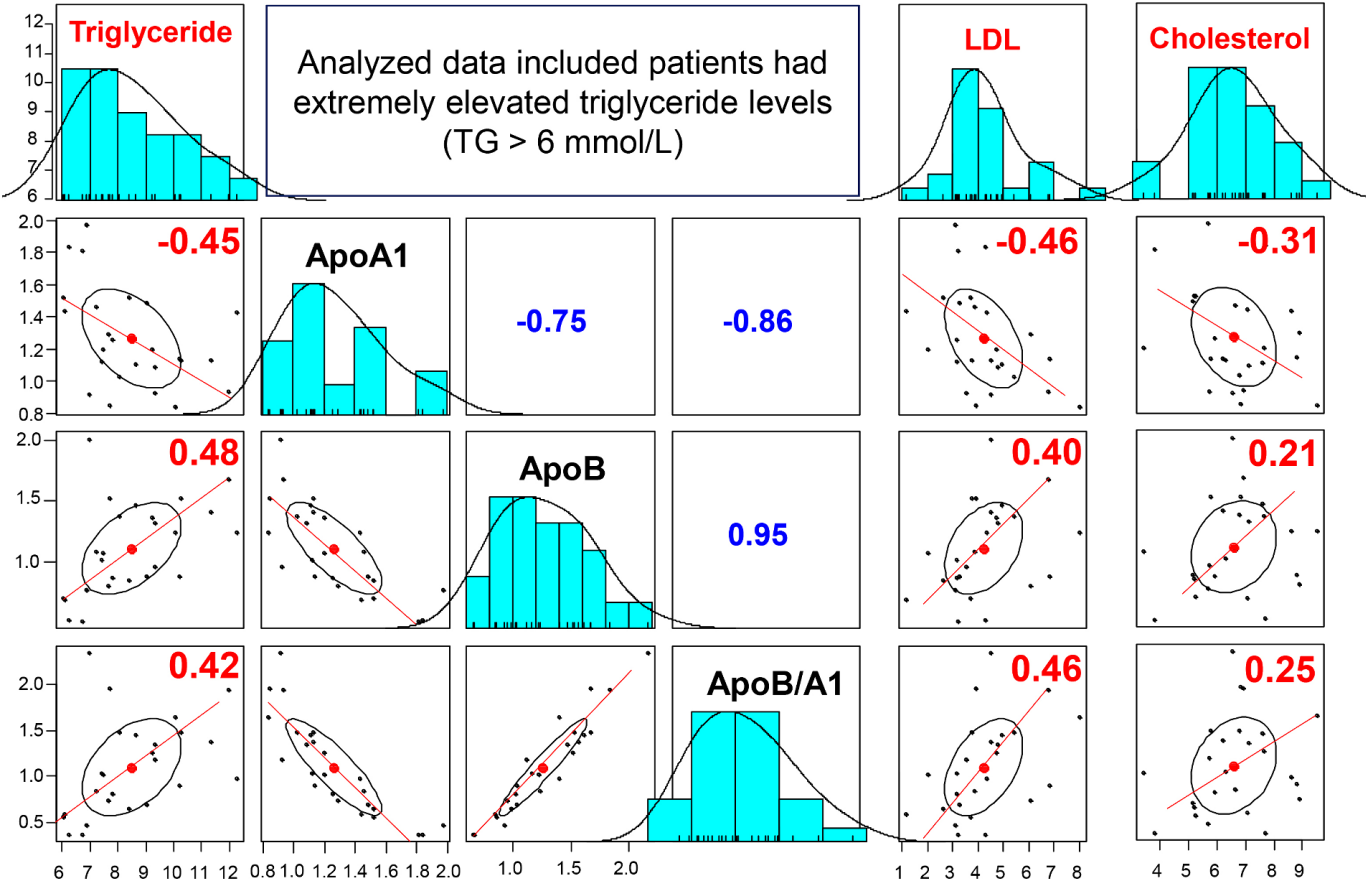
Correlation between apoA-I, apoB, apoB/apoA-I ratio and conventional lipid in IS patients with triglyceride >6 mmol/L: The correlation coefficients between two variables (correlations between apoB, apoA-I, apoB/apoA-I with triglyceride, LDL, Cholesterol) were calculated by using spearman’s rank correlation coefficient. Spearman’s rho (rho) values are given.

We further analyzed the correlation between serum levels of triglycerides, LDL, cholesterol and apo-A1, apoB, apoB/apoA-I ratio, as illustrated in Figure 5. Our findings revealed significant correlations between apoA-I levels and triglyceride, LDL, and cholesterol, with rho values of −0.45, 0.46, and −0.31, respectively (although not significant correlation for all IS patients as previously reported). Furthermore, a stronger correlation was observed between apoB levels, the apoB/apoA-I ratio and triglyceride, LDL levels in this sub-analysis.

### Association of apoA-I, apoB levels, and apoB/apoA-I ratio with arterial stenosis

**Figure 6:**
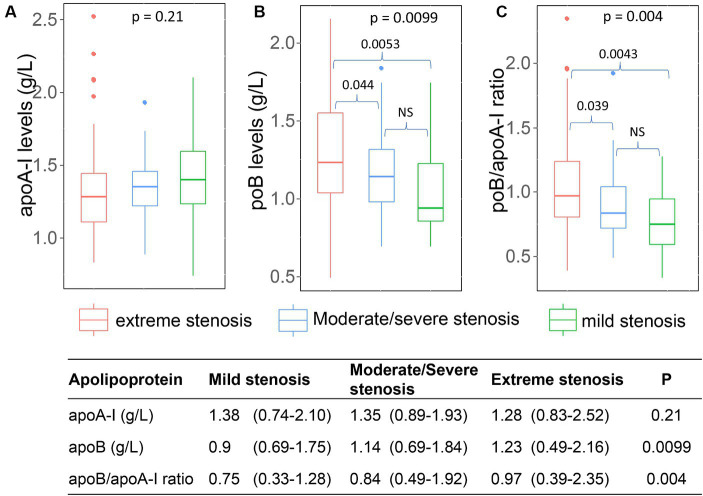
Association of apoA-I, apoB, apoB/apoA-I ratio with the arterial stenosis levels: **(A)** apoA-I; **(B)** apoB; **(C)** apoB/apoA-I ratio. Box-plots illustrate median values with range (min-max) and outliers; NS, not significant; *p*-values were calculated by Wilcoxon or Kruskal Wallis tests where appropriate.

The mild stenosis group exhibited the highest levels of apoA-I [median and range: 1.38 (0.74–2.1)], followed by the severe stenosis [median and range: 1.35 (0.89–1.93)] group and the occlusion group [median and range: 1.28 (0.83–2.52)], although these differences did not reach statistical significance. Conversely, the lowest concentration of apoB and apoB/apoA-I ratio was observed in the mild stenosis group [ApoB: 0.9 (0.69–1.75); ApoB/ApoA-I: 0.75 (0.33–1.28)], followed by the severe stenosis group [ApoB: 1.14 (0.69–1.84); ApoB/ApoA-I: 0.84 (0.49–1.92)]. The highest levels were recorded in the occlusion group [ApoB: 1.23 (0.49–2.16); ApoB/ApoA-I: 0.97 (0.39–2.35)], with statistical significance as depicted in Figure 6.

Upon detailed analysis in the subset of 25 cases with triglyceride levels exceeding 6 mmol/L, data regarding arterial stenosis were accessible for 17 patients. This analysis revealed that out of the 17 patients, 14 (82%) exhibited extreme stenosis, while two patients had mild stenosis, and one patient had severe stenosis.

**Figure 7:**
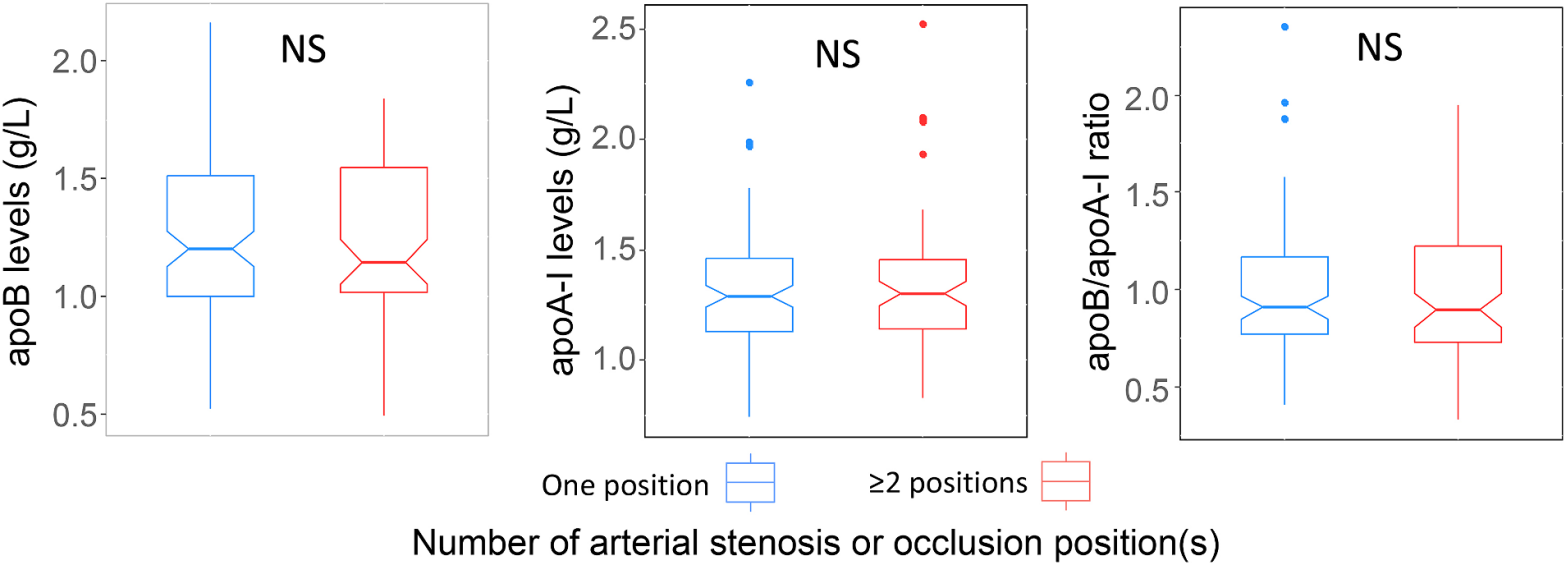
Association of apoA-I, apoB, apoB/apoA-I ratio with number of stenosis or occlusion sites: Box-plots illustrate median values with range (min-max) and outliers; NS, not significant; *p*-values were calculated by Wilcoxon tests.

Within the ICAS group, the serum levels of apoA-I, apoB and the apoB/apoA-I ratio in patients with single-site stenosis/occlusion were not different from patients with multi-site stenosis/occlusion (Figure 7). We could not generate the analyses for ECAS and SAO group because of missing data (ECAS) or unavailable data (SAO).

### Association of apoB/apoA-I ratio with and arterial stenosis severity and IS subtypes

**Figure 8:**
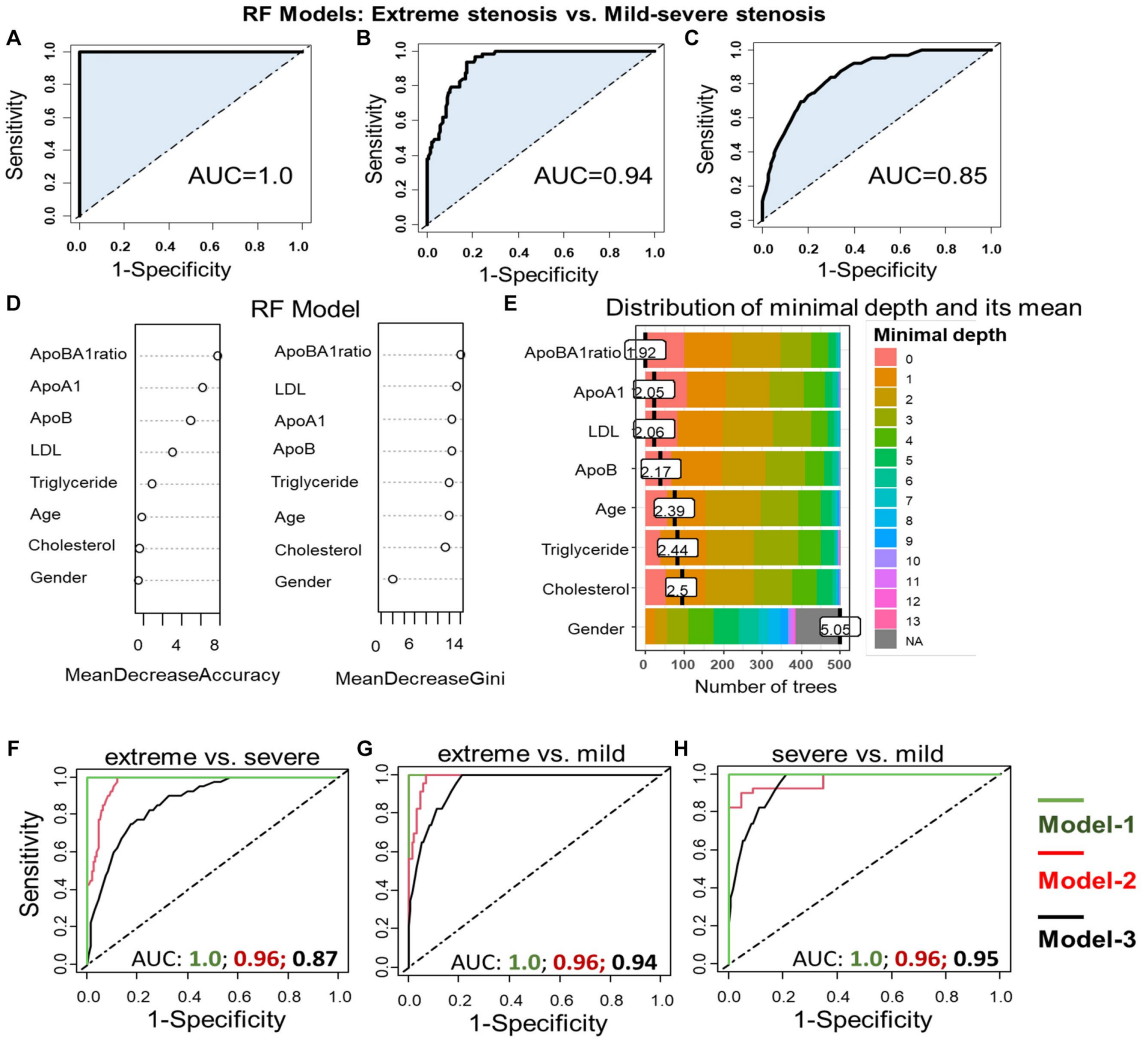
Association apoB/apoA-I ratio with arterial stenosis severity: Performance of different RF models in differentiating extreme stenosis from mild–severe stenosis group **(A–C)**. RF model-1 included variables: apoB/apoA-I ratio, apoA-I, apoB, LDL, cholesterol, triglyceride, age, and gender; RF model-2 included variables: apoB/apoA-I ratio, age, and gender; RF model-3 included only apoB/apoA-I ratio. The AUC values were accordingly presented under the FR model-1 **(A)**, the RF model-2 **(B)**, and the model-3 **(C)**. Variable importance plot **(D)** and plot of distribution of minimal depth **(E)**. The similar RF models were constructed to differentiate extreme from severe stenosis **(F)**; extreme from mild stenosis **(G)**; severe from mild stenosis **(H)**.

We utilized different random forest (RF) models to assess factors associated with the severity of arterial stenosis in IS patients. The model-1 included variables apoB/apoA-I ratio, apoA-I, apoB, LDL, cholesterol, triglyceride, age, and gender. The model-2 included variables as apoB/apoA-I ratio, age, and gender. The model-3 included only apoB/apoA-I ratio. The results were illustrated in Figure 8 and we have shown that that all RF models performed well in predicting the degree or severity of arterial stenosis. For instance, in models predicting extreme stenosis from mild–severe stenosis, the AUC values were 1.0 for model-1 (Figure 8A); 0.94 for model-2 (Figure 8B); 0.85 for model-3 (Figure 8C). In addition, to determine the most influential parameter in the model-1, we constructed plots of variable importance and distribution of minimal depth through tree modeling. The results revealed that the apoB/apoA-I ratio played the most significant role in predicting extreme stenosis from mild–severe stenosis compared to other variables as indicated by their low mean minimal depth and high values in both mean decrease accuracy and mean decrease gini values (Figures 8D,E). Similar pattern was observed in the other RF models (extreme vs. severe; extreme vs. mild; severe vs. mild) shown in Figures 8F–H.

**Figure 9:**
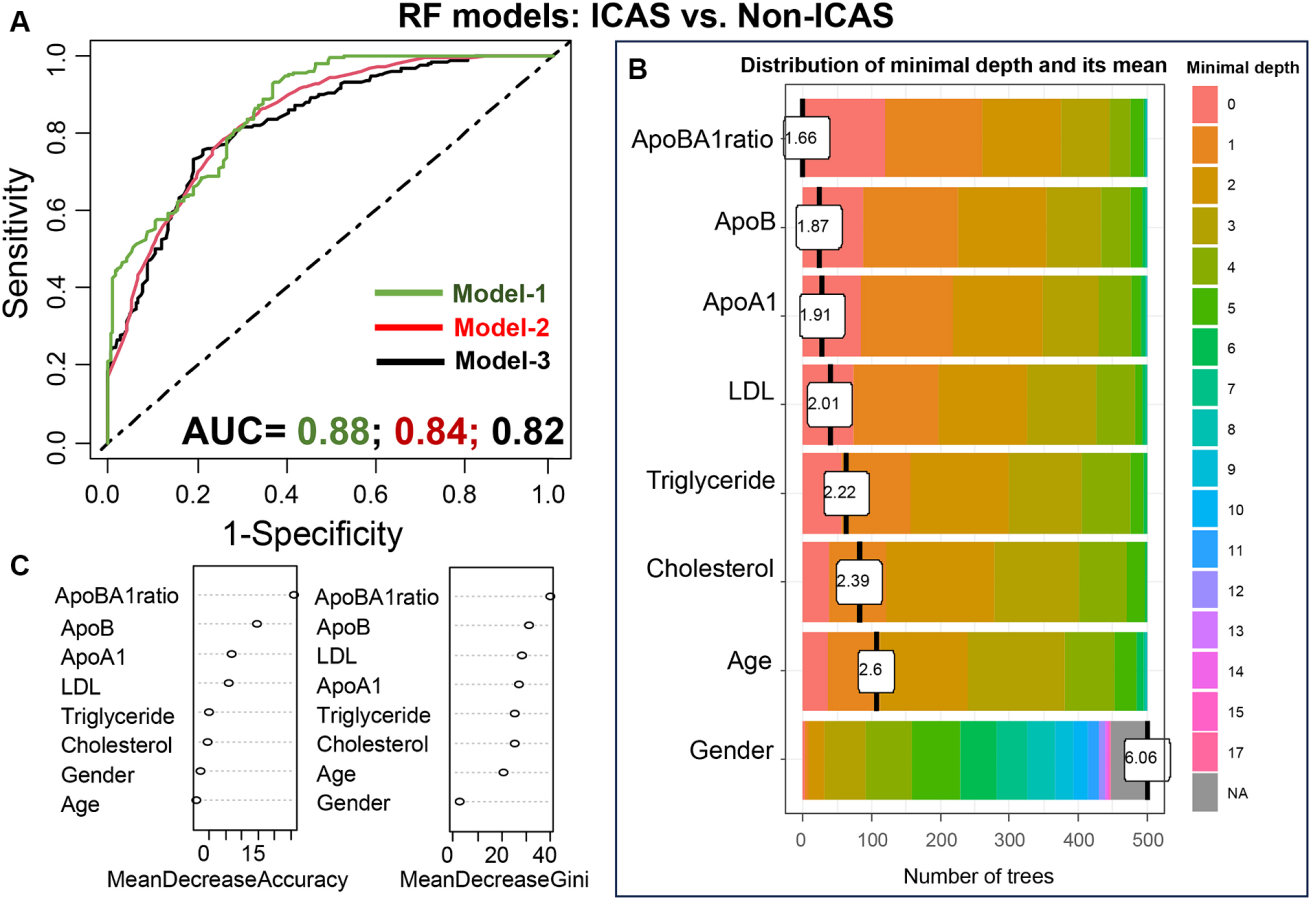
Performance of the RF models in differentiating ICAS from non-ICAS patients: RF model-1 included variables: apoB/apoA-I ratio, apoA-I, apoB, LDL, cholesterol, triglyceride, age, and gender; RF model-2 included variables: apoB/apoA-I ratio, age, and gender; RF model-3 included only apoB/apoA-I ratio. The AUC values were presented under the FR model-1/-2/-3 **(A)**; Variable importance plot **(B)** and plot of distribution of minimal depth **(C)**.

To assess the significant predictors of IS subtypes, we constructed RF models, incorporating multiple variables (models 1 and 2) or single variable (model 3, as previously described). The results of our analysis are depicted in Figure 9, illustrating the prediction of ICAS patients from non-ICAS patients (ECAS+SAO), with AUC values of 0.88 (model 1), 0.84 (model 2), and 0.82 (model 3). Plots of variable importance and distribution of minimal depth revealed that the apoB/apoA-I ratio was the most influential variable compared to others such as apoA-I, apoB, LDL.

## Discussion

Ischemic stroke remains a significant global health concern, with arterial atherosclerosis playing a central role in its pathogenesis. Atherosclerosis, a chronic inflammatory disease characterized by the accumulation of lipid-rich plaques within arterial walls, underlies the majority of IS cases. Apolipoproteins, essential components of lipoproteins, are intimately involved in lipid metabolism and transport, exerting both pro-atherogenic (apoB) and anti-atherogenic (apoA-I) effects (17). In our study, we observed significant differences in apoA-I and apoB levels across IS subtypes, with ICAS patients exhibiting distinct alterations compared to ECAS and SAO patients, highlighting the potential involvement of these apolipoproteins in the progression of intracranial atherosclerosis. These findings corroborate previous evidences, implicating low serum levels of apoA-I and high levels of apoB in the pathogenesis of atherosclerosis in ICAS (28, 29).

The formation of atherosclerotic lesions varies significantly among different IS subtypes, including ICAS, ECAS, and SAO, each presenting distinct pathophysiological mechanisms and clinical implications [reviewed in (9)]. In ICAS, atherosclerotic plaques predominantly affect the large arteries within the intracranial vasculature, such as the middle cerebral artery or the basilar artery. These lesions typically arise from chronic inflammation and the buildup of lipid deposits within the arterial wall, leading to luminal stenosis and subsequent ischemic events (9, 30). On the other hand, ECAS involves atherosclerotic changes in the extracranial arteries, including the carotid and vertebral arteries. Risk factors such as hypertension, smoking, and dyslipidemia contribute to the development of ECAS, with plaque rupture and thrombosis being common mechanisms underlying IS in these patients (31). SAO, meanwhile, is characterized by the occlusion of small perforating arteries deep within the brain parenchyma. Unlike ICAS and ECAS, SAO is often associated with hypertensive arteriopathy and lipohyalinosis, leading to lacunar infarcts and small vessel disease. While all three types of atherosclerotic lesions can result in IS, ICAS and ECAS are typically considered more severe due to their potential for large infarcts and hemispheric involvement. SAO, although associated with a higher risk of recurrent strokes, often presents with smaller lesions and milder clinical manifestations (32–34).

The significance of apoA-I and apoB in ICAS can be explained in detail by their roles in atherosclerosis, the underlying pathology of ICAS. ApoA-I is the major protein component of HDL particles, known for its role in reverse cholesterol transport, which removes excess cholesterol from peripheral tissues and transports it to the liver for excretion (35, 36). Conversely, apoB is a key component of atherogenic lipoproteins such as LDL and very-low-density lipoprotein (VLDL), which transport cholesterol to peripheral tissues and contribute to the vascular inflammation, which are key mechanisms in ICAS. In this line, elevated levels of apoB and LDL cholesterol are well-established risk factors for atherosclerosis and cardiovascular disease (17, 37). In the context of ICAS, studies have shown associations between low levels of apoA-I and high levels of apoB with the presence and severity of ICAS, indicating their potential as biomarkers for ICAS risk stratification (23, 38).

The apoB/apoA-I ratio reflects the equilibrium between atherogenic and antiatherogenic lipoproteins in the bloodstream (19, 20, 39), with a higher ratio being linked to an elevated risk of stroke (23, 40). These findings suggest that the ApoB/ApoA-I ratio might serve as a superior indicator of stroke risk compared to other lipid profiles and their ratios (40). Consistent with this notion, our study demonstrates clear and significant associations between the apoB/apoA-I ratio and ICAS, emphasizing the role of dysregulated lipid metabolism in the pathogenesis of ICAS and providing additional evidence supporting the importance of the apoB/apoA-I ratio in IS subtyping (38, 41, 42). Recent study revealed significant associations between the apoB/apoA-I ratio and ICAS, while no such associations were observed with ECAS (42). The significant associations between the apoB/apoA-I ratio and ICAS can be explained by the role of apoA-I as a marker of antioxidant properties, with intracranial arteries exhibiting greater activity of antioxidant enzymes than extracranial arteries (43). Elevated levels of apoA-I and reduced levels of apoB, resulting in a higher apoB/apoA-I ratio, lead to increased activity of antioxidant enzymes in intracranial arteries, potentially contributing to their greater resistance to atherogenesis (43).

In our study, patients with ICAS had a lower value of apoA-I compared with ECAS and SAO patients. Therefore, reduced antioxidant activities in ICAS might explain our findings of an association of the apoB/apoAI ratio with ICAS but not with ECAS. Furthermore, we have revealed significant differences in apoB/apoA-I ratio across different degrees of arterial stenosis severity. While the mild stenosis group exhibited the highest apoA-I levels, the occlusion group showed the lowest. Conversely, both apoB concentration and the apoB/apoA-I ratio followed an opposite pattern, with the mild stenosis group displaying the lowest values and the occlusion group exhibiting the highest.

Despite the comprehensive analyses and investigation, there are some limitations that should be acknowledged. Firstly, although controlling for various demographic and clinical factors, residual confounding remains a possibility. Factors such as diet, physical activity, medication use, and comorbidities could confound the associations between apolipoproteins and IS subtypes, which were not fully accounted for in the current study. Secondly, our sample predominantly comprised patients from a single medical unit, limiting the generalizability of our findings to other populations.

## Conclusion

Our study contributes to understand the potential contribution of apoA-I and apoB to the pathogenesis of ischemic stroke subtypes and highlights the potential clinical relevance of the apoB/apoA-I ratio in ICAS risk stratification and stenosis severity. Further studies are warranted to validate these findings and enhance their clinical applicability.

## Data availability statement

The original contributions presented in the study are included in the article/supplementary material, further inquiries can be directed to the corresponding authors.

## Ethics statement

The studies involving humans were approved by Institutional Review Board of the 108 Military Central Hospital, Hanoi, Vietnam. The studies were conducted in accordance with the local legislation and institutional requirements. Written informed consent for participation was not required from the participants or the participants’ legal guardians/next of kin due to the observational nature of the study.

## Author contributions

NVT: Conceptualization, Data curation, Formal analysis, Writing – review & editing. NHN: Conceptualization, Resources, Writing – review & editing. PQH: Data curation, Investigation, Resources, Writing – review & editing. NTY: Data curation, Investigation, Writing – review & editing. NXH: Data curation, Formal analysis, Visualization, Writing – original draft, Writing – review & editing. NCT: Conceptualization, Data curation, Investigation, Methodology, Project administration, Writing – review & editing.

## Footnotes

^1^ http://www.r-project.org
